# Machine learning-based analyzing earthquake-induced slope displacement

**DOI:** 10.1371/journal.pone.0314977

**Published:** 2025-02-06

**Authors:** Jiyu Wang, Niaz Muhammad Shahani, Xigui Zheng, Jiang Hongwei, Xin Wei

**Affiliations:** School of Mines, China University of Mining and Technology, Xuzhou, Jiangsu Province, China; Guizhou University, CHINA

## Abstract

Accurately evaluating earthquake-induced slope displacement is a key factor for designing slopes that can effectively respond to seismic activity. This study evaluates the capabilities of various machine learning models, including artificial neural network (ANN), support vector machine (SVM), random forest (RF), and extreme gradient boosting (XGBoost) in analyzing earthquake-induced slope displacement. A dataset of 45 samples was used, with 70% allocated for training and 30% for testing. To improve model robustness, repeated 5-fold cross-validation was applied. Among the models, XGBoost demonstrated superior predictive accuracy, with an R^2^ value of 0.99 on both the train and test data, outperforming ANN, SVM, and RF, which had R^2^ values of 0.63 and 0.80, 0.87 and 0.86, 0.94 and 0.87 on the train and test data, respectively. Sensitivity analysis identified maximum horizontal acceleration (kmax = 0.714) as the most influential factor in slope displacement. The findings suggest that the XGBoost model developed in this study is highly effective in predicting earthquake-induced slope displacement, offering valuable insights for early warning systems and slope stability management.

## 1. Introduction

Evaluating the stability of earth infrastructure, including slopes, in the context of earthquakes is crucial due to the significant environmental, financial, and human impacts posed by seismic events [1]. Slopes with a magnitude of 4 or higher are prone to partial failure, while those experiencing a magnitude of 6 or more may face complete instability [2, 3]. Factors such as slope configuration, ground vibrations, and material properties play a crucial role in earthquake-induced slope failures [1]. Various methods have been proposed to model earthquake-induced slope displacement, including Saygili and Rathje developed an empirical model for predicting slope movement during an earthquake [4], while Lin and Whitman introduced a procedure to estimate permanent displacement caused by ground accelerations [5]. More advanced analyses, such as probabilistic assessments by Rathje and Raugali [6], and reliability analyses by Refice and Capolongo [7] have also contributed to understanding slope stability under earthquake loads. Al-Homond and Tahtamoin considered uncertainties in earthquake-induced movement and 3D slope stability [8]. Yuan et al. carefully investigated the effects of acceleration on sliding surfaces during earthquake-induced landslides [9]. Babanouri and Dehghani studied shear strains and failure probabilities for large, potentially slide-prone slopes during design earthquakes [10]. Bray and Travasarou employed a stochastic, complementary sliding-block framework to create a semi-empirical correlation for predicting displacement [11]. They also presented a straightforward approach to calculate pseudo-static benchmarks based on spectral acceleration, permissible movement, and earthquake magnitude [12]. To assess slope stability during earthquakes, Jibson [13] categorized evaluation methods into three general groups: “(1) stress-deformation analysis, (2) permanent-displacement analysis, and (3) pseudo-static analysis”. Bojadjieva et al. studied risk assessment and landslide hazards in Skopje, Macedonia, considering various water conditions and earthquake structures [14]. To enhance prediction accuracy, machine learning (ML) has emerged as a powerful tool, complementing traditional techniques like Monte Carlo simulation (MCS) in assessing slope failure risks [15–17].

ML has increasingly become a robust tool for analyzing various complex phenomena, including earthquake-induced displacement of slopes. Recent studies have demonstrated its effectiveness in diverse applications within geotechnical engineering and beyond. For instance, Cho et al. [18] emphasized the importance of nonlinear finite element analysis in generating displacement hazard curves for slopes affected by earthquakes, highlighting its role in improving predictive accuracy. In a related context, Lis-Gutiérrez et al. [19] explored the prediction of spending levels among displaced populations using random forest (RF) and support vector machine (SVM), underscoring the need to understand key variables driving these predictions. In slope stability, Dong et al. [20] introduced a real-time wireless monitoring system integrated with an autoregressive recurrent networks (DeepAR) model for predicting the deformation of unstable slopes. This approach has proven effective in ensuring safety during construction and providing accurate predictions during ongoing operations. Similarly, Durante et al. [21] applied the RF model to predict lateral spreading patterns, achieving high accuracy in identifying and classifying displacement occurrences. Daribayev et al. [22] predicted oil recovery, highlighting the importance of robust algorithms as reservoir models become increasingly complex. In addition, Melchor-Leal et al. [23] proposed an extreme learning machine (ELM) algorithm for characterizing force profiles in thermostatic bimetallic strips, demonstrating its predictive power. Thackway et al. [24] developed a tree-based model to predict gentrification trends in Sydney, demonstrating the potential of such techniques in urban planning and neighborhood analysis. Xu et al. [25] evaluated slope stability by developing RF, gradient boosting decision tree (GBDT), extreme gradient boosting (XGBoost), and light gradient boosting machine (LightGBM) for dynamic assessment based on multi-source monitoring data.

This study aims to evaluate the performance of various ML algorithms, including ANN, SVM, RF, and XGBoost, in predicting earthquake-induced slope displacement. The approach involves first collecting data on earthquake-induced slope displacement from existing literature. By comparing these models using different performance metrics, the study seeks to identify the most effective approach for assessing slope failure risk due to earthquake-induced displacements.

## 2. Dataset

The dataset used in this study was obtained from the work of Ferentinou et al. [26], encompassing a total of 45 original datasets. These datasets provide valuable insights into various parameters related to the study’s objectives. The input parameters considered in the analysis include height (H) in (m), unit specific weight (γ) in kN/m, cohesion (C) in kPa, angle of internal friction (φ), significant duration of shaking (D5–95), maximum horizontal acceleration (kmax), and return displacement (*u*) in cm. Based on Ferentinou et al. [26], the slope characteristics crucial for stability assessment include factors like H (m), ɣ (kN/m^3^), C (kPa), and Φ *(*^*o*^*)*. These properties, combined with external influences like pore water pressure and earthquake forces, contribute to different deformation mechanisms, such as circular or wedge failures. The study highlights that these factors directly impact the slope’s response under seismic conditions. In parallel, ML models are employed to predict earthquake-induced slope displacement, using these input factors to estimate the likelihood and extent of failure under both static and dynamic conditions. The dataset used in this study is detailed in Table 1.

**Table 1:** The dataset used in this study.

Dataset	H (m)	ɣ (kN/m^3^)	C (kPa)	Φ *(*^*o*^*)*	D5–95	kmax	*u* (cm)
1	12	22	8	35	7.9	0.24	0.25
2	12	22	8	35	8.35	0.2	0.06
3	12	22	8	35	9.55	0.13	0.0008
4	12	22	8	35	11.1	0.33	2.7
5	12	22	8	35	11.5	0.27	0.82
…	…	…	…	….	…	…	…
41	6	21	5	35	11.5	0.27	0.26
42	6	21	5	35	12.7	0.18	0.006
43	6	21	5	35	16	0.45	9.24
44	6	21	5	35	16.4	0.37	3.24
45	6	21	5	35	17.65	0.24	0.16

**Fig 1:**
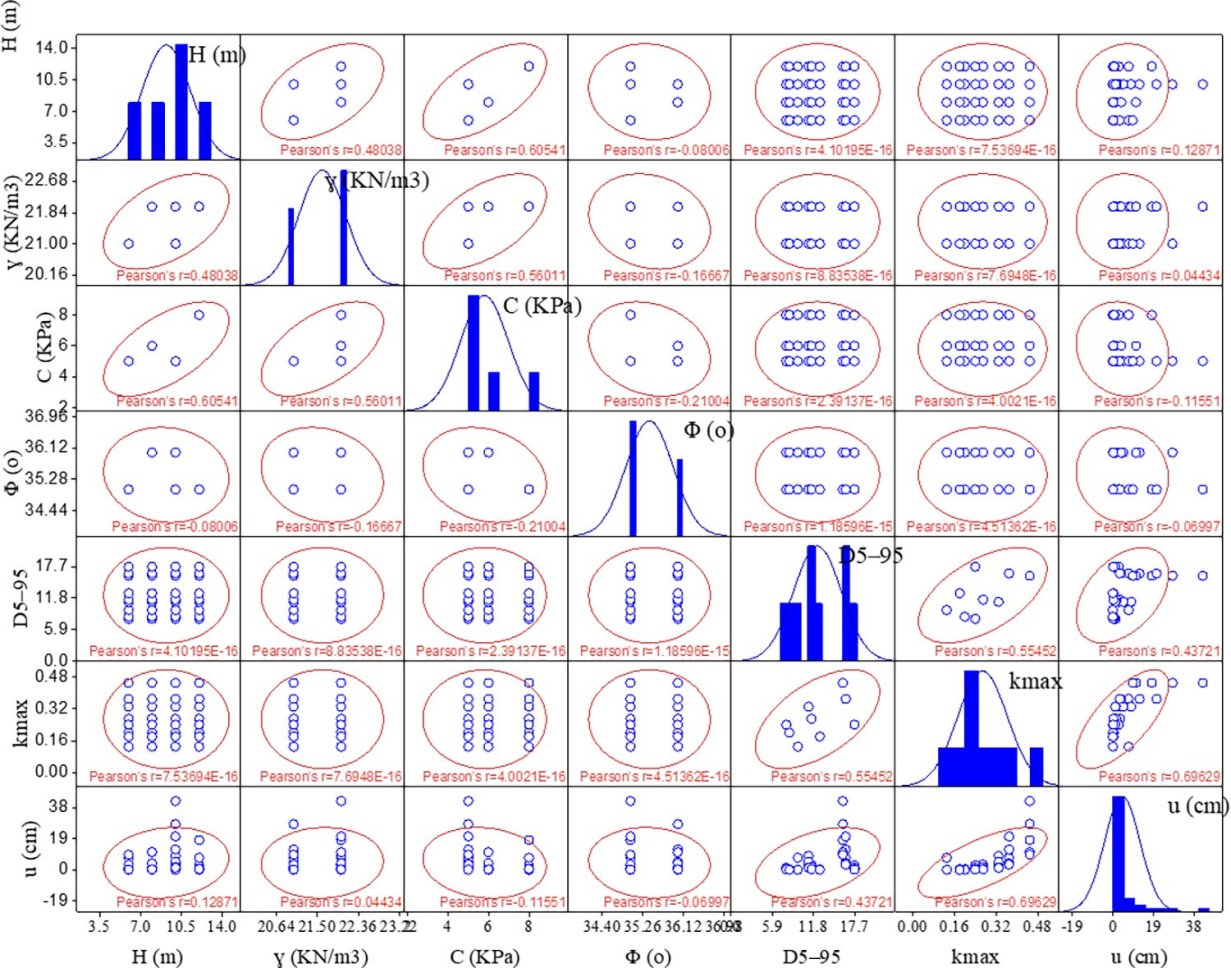
Statistical distribution of various parameters in the dataset used in this study.

Fig 1 illustrates the statistical distribution of different input parameters and the *u*. It is important to highlight that most of the parameters are not highly correlated with one another, which allows for a comprehensive examination of all parameters in predicting the *u*. Notably, the maximum kmax shows a moderate correlation with the *u*. The correlation analysis of the entire dataset is also presented. A correlation coefficient near 1 defines a strong positive correlation between parameters, while a negative correlation coefficient near -1 defines a strong inverse relationship between parameters. On the other hand, a correlation coefficient of zero indicates no correlation between the parameters. In this study, apart from the kmax, none of the input parameters exhibit a significant correlation with the *u*.

The analysis conducted in this study examined the relationship between input variables and the output variable, *u*, in the dataset. Input variables were selected based on their correlation with the target variable, prioritizing those with stronger correlations for their direct impact on model performance. Weaker correlated variables were also included to capture potential non-linear interactions. This approach balances predictive accuracy and generalizability by incorporating both statistically significant and practically relevant factors, thereby enhancing the model’s robustness through careful parameter selection.

## 3. Methods

**Fig 2:**
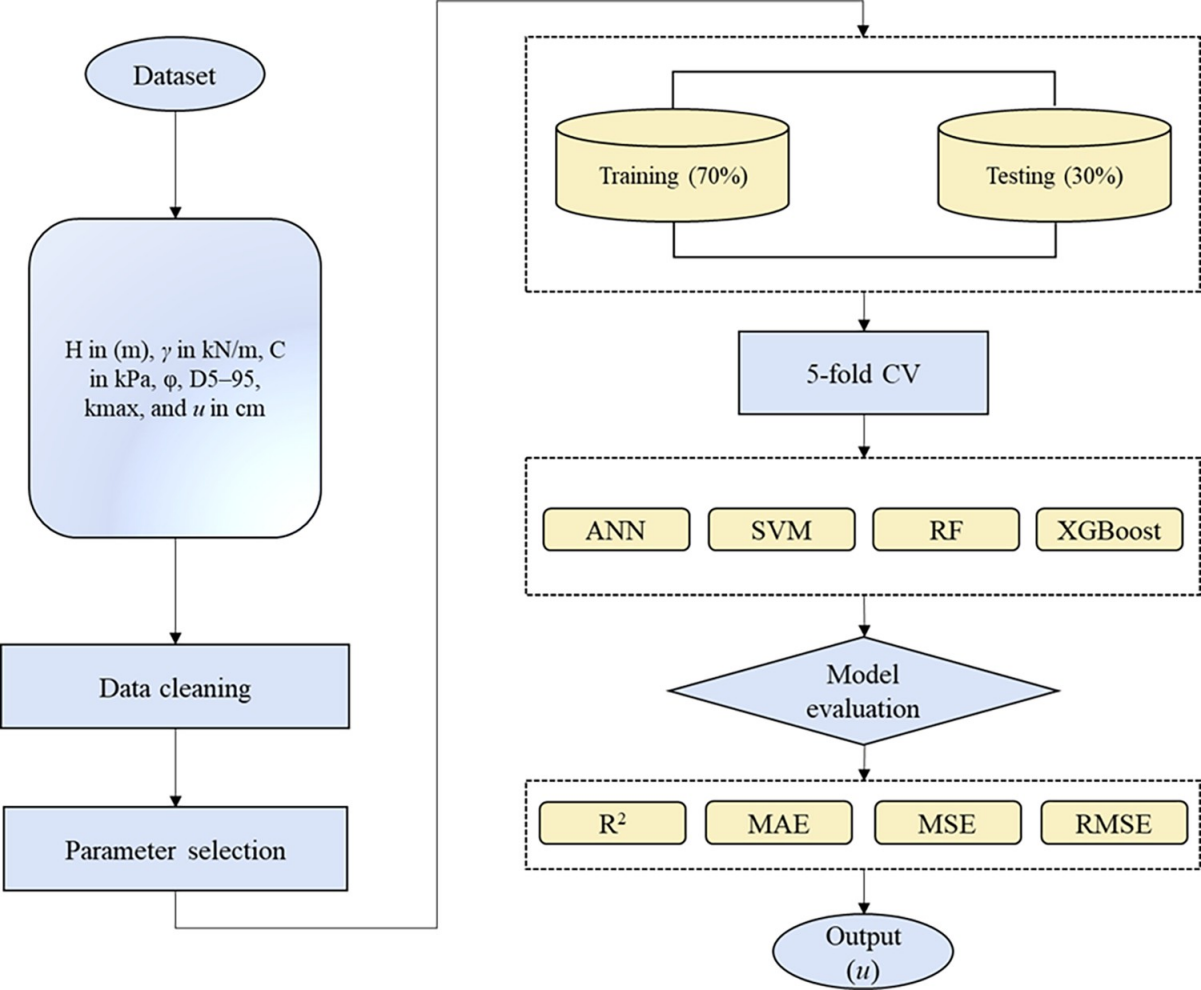
Flowchart of the proposed methodology.

This study utilizes various ML models, particularly ANN, SVM, RF, and XGBoost, to analyze earthquake-induced slope displacement *u*. The predictive performances of these models are thoroughly compared from various perspectives. The proposed methodology is outlined in Fig 2. Initially, raw slope displacement data is collected from a genuine rock mechanics project. The data is then organized, with input and output values arranged to facilitate the execution and relationship analysis of the three frameworks. The input parameters include H in (m), γ in kN/m, C in kPa, φ, D5–95, kmax, and *u* in cm. The original data is randomly split into a training set with 70% and a test set with 30%, ensuring consistency in slope displacement data during the split. A 5-fold cross-validation is then performed on the training dataset to determine robust hyperparameters for the ANN, SVM, RF, and XGBoost models. These models are trained using the dataset with the tuned hyperparameters. The test set is used to evaluate model performance through various metrics, including coefficient of determination (R^2^), mean absolute error (MAE), mean squared error (MSE), and root mean squared error (RMSE). Finally, models are assessed, and the best-performing model is selected for implementation.

### 3.1. Artificial neural network

ANN is a crucial ML mechanism known for its remarkable self-learning, self-instructing, and self-adapting capabilities. These attributes have led to extensive research and successful application in addressing various real-life challenges [27]. The term "neural” in ANNs reflects their inspiration from the human brain’s learning processes. ANNs offer a robust approach for statisticians to uncover intricate and numerous patterns in real-world problems. They excel at predicting complex, multi-directional relationships between input and output parameters [28]. ANNs are versatile and can be utilized across various fields for classification, regression, prediction, and solving complex problems, including nonlinear issues [29]. ANNs are versatile numerical analysis tools applicable to various problems, including “speech recognition, pattern classification, adaptive interfaces between complex physical systems and humans, clustering, prediction, and forecasting” [30]. In real-world scenarios, the alteration of associative states among different neurons, as described by Hebb’s Learning Rule, can occur. The outcomes are influenced by previous perception models and computational neuron models with additional weighting [31]. Currently, multilayer perceptrons (MLPs), which are based on perceptrons and the number of neurons, have been implemented with high standards of perceptron connections. Researchers frequently use these models to tackle complex learning problems across various domains [32].

### 3.2. Support vector machine

In 1997, SVM was originated by Vapnik et al. [33], which is a form of supervised learning. SVM can be adapted for both classification and regression tasks. In the context of regression, support vector regression (SVR) is used to identify the optimal hyperplane that best predicts continuous output values while maintaining a specified margin of tolerance. SVR is particularly effective for high-dimensional datasets, offering robust resistance to overfitting, even when the number of features exceeds the number of samples. By leveraging kernel functions, SVR can model complex, nonlinear relationships within the data, making it a highly versatile and powerful tool for regression analysis. SVM uses high-dimensional feature spaces, and the prediction function is constructed using Vapnik’s *ε*-insensitive loss function and kernel functions [34].

### 3.3. Random forest

RF is an ELM method that constructs multiple decision trees to predict results for regression tasks. During training, each tree is built using a randomly selected subset of both features and data points, which presents variability among the trees and improves the overall model diversity. The final prediction is obtained by averaging the predictions from all individual trees, which helps to counteract the overfitting often associated with single decision trees. This aggregation process leads to a robust and stable model that achieves high predictive accuracy while being less sensitive to noise and fluctuations in the data. This approach is effective in handling complex regression problems due to its ability to generalize well across different datasets.

### 3.4. Extreme gradient boosting

**Fig 3:**
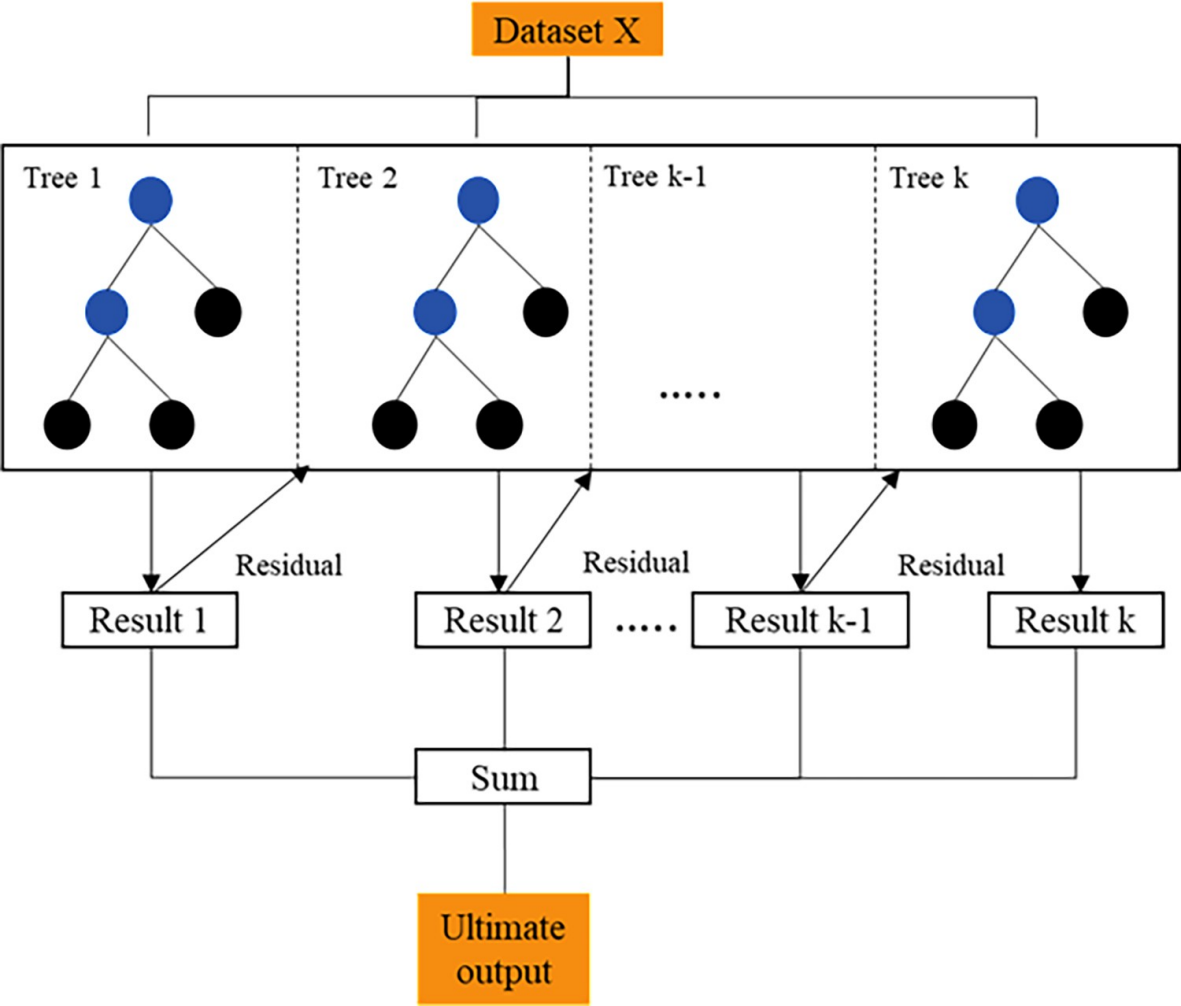
The architecture of the XGBoost model.

XGBoost, or extreme gradient boosting, is an ensemble learning algorithm that enhances machine learning techniques through statistical boosting methods [35]. It builds on simple classification and regression trees (CARTs) by integrating multiple trees into a consensus prediction framework. Unlike constructing a single tree, boosting improves model precision by sequentially adding trees, each addressing the residuals of previous trees. This process iteratively refines predictions by minimizing errors from prior trees. The iterative nature of XGBoost can be viewed as a form of gradient descent, where each new tree is introduced to reduce the residuals of the previous trees [36]. The expansion of new trees continues until either the maximum number of trees is reached or the training error stabilizes, achieving a pre-defined target. To further enhance estimation precision and computational efficiency, XGBoost incorporates random sampling, known as probabilistic slope boosting [37]. In this approach, a random subset of the training data is used for each tree, rather than the full dataset, which helps to improve model performance without overfitting. XGBoost employs second-order loss function estimation, which accelerates convergence compared to traditional gradient boosting machines (GBMs). Its advanced capabilities have made it a powerful tool in various applications, including the analysis of gene expression data in mining research [38]. The XGBoost algorithm builds trees in a level-wise (depth-wise) manner, as depicted in Fig 3.

### 3.5. Evaluation criterion

To accurately evaluate the performance of ANN, SVM, RF, and XGBoost models, the following evaluation criteria (Eqs (1) to (4)) are used to assess the relationship between measured and predicted values. These criteria include the R^2^, MAE, MSE, and RMSE.


$$R^{2}=1-\frac{\sum_{i=1}^{n}{\left(X-X^{\prime }\right)}^{2}}{\sum_{i=1}^{n}{\left(X-X\prime \prime \right)}^{2}}$$ Eq (1)

$$MAE=\sum_{i=1}^{n}⎸X\prime \prime -X⎸$$ Eq (2)

$$MSE=\frac{1}{T}\sum_{i=1}^{n}{\left(X-X\prime \prime \right)}^{2}$$ Eq (3)

$$RMSE=\sqrt{\frac{1}{T}\sum_{i=1}^{n}{\left(X-X\prime \prime \right)}^{2}}$$ Eq (4)

Here, *X*′ and *X*′′ show predicted values and the mean values, respectively. *T* denotes the total number of datasets and *X* represents the actual values.

### 3.6. Hyperparameter tunning

Optimizing hyperparameters is crucial for enhancing the accuracy of ML models. In this study, hyperparameters were adjusted using the cross-validation method and data normalization, rather than being set manually due to the limited dataset on earthquake-induced slope displacements. The data is divided into training and testing to enhance the models’ performance. The training dataset is used to evaluate the model, and the testing dataset assesses the final performance [39]. To address potential non-linearity in the dataset, the k-fold cross-validation technique is used. In this method, the data is divided into *k* segments, with each segment serving as a test set while the remaining *k*−1segments are used for training. This approach enables multiple evaluations, yielding metrics such as R^2^, MAE, MSE, and RMSE, as well as allowing for the calculation of average and standard deviation values for these metrics.

### 3.7. Grid Search Cross-Validation(CV)

**Fig 4:**
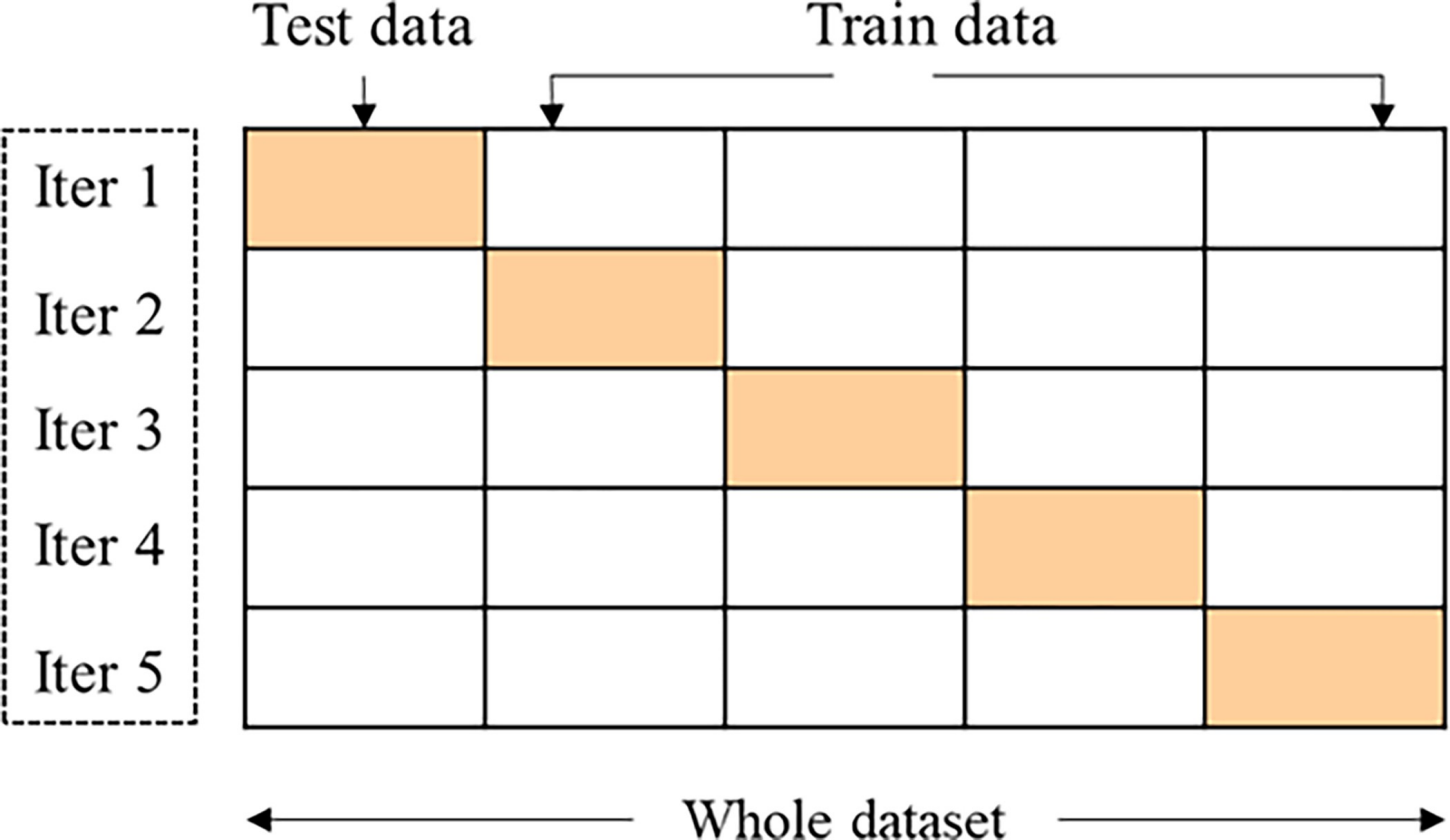
5-fold cross-validation.

A grid search method was employed using the GridSearchCV() function from the scikit-learn library in Python to optimize the hyperparameters of the models [40]. This method systematically explores all possible combinations of hyperparameters within the defined search space and computes cross-validation scores for each combination. In this study, 5-fold cross-validation with repeated random sampling was used, as shown in Fig 4. This approach helps mitigate the risk of overfitting by providing an average performance score across the folds, rather than relying solely on the best score from one-fold. While GridSearchCV() identifies the combination of best hyperparameters that yield the highest cross-validated score, it is important to consider the performance across all folds to avoid bias in model evaluation. Thus, the results from each fold are reported, ensuring that the identified optimal hyperparameters are not solely based on a single fold but reflect consistent performance across the dataset. All other parameters in the Python environment were left at their default settings during the analysis.

## 4. Analysis of results and discussion

The analysis revealed that training the ANN, SVM, RF, and XGBoost models on the entire dataset may lead to overfitting. Specifically, while the models perform well on the training data, they may struggle to accurately predict new, unseen data. To mitigate this issue, the slope displacement dataset was split into 70% for training and 30% for model testing. To avoid localized bias, the data was randomly shuffled before splitting. The models were then trained on the training dataset and validated using the testing dataset.

Determining the number of neurons in the hidden layers of the ANN involved a coarse search method followed by a refined search approach. Three configurations of hidden layers were tested, as shown in Table 2. The number of hidden neurons was initially set using a binary search to values of 32, 64, and 128 neurons. It was observed that, with appropriate parameters, the ANN models produced reasonably optimal results. For example, an ANN model with two hidden layers, each containing 32 neurons, using the rectified linear unit (ReLU) activation function, the RMSProp optimizer, and trained for 200 epochs, demonstrated effective performance.

**Table 2:** Performance metrics versus the number of neurons in the ANN model.

No.	Model	Hidden Layers	Neurons	R^2^	MAE	MSE	RMSE
1	ANN	1	32	0.82	1.81	4.43	2.11
			64	0.83	1.74	4.07	2.02
			128	0.88	1.38	2.79	1.67
2		2	32	0.98	0.44	0.35	0.59
			64	0.98	0.46	0.50	0.70
			128	0.98	0.49	0.39	0.63
3		3	32	0.97	0.82	0.81	0.90
			64	0.97	0.57	0.64	0.80
			128	0.97	0.53	0.70	0.84

Table 2 shows the performance of the ANN model with varying neuron numbers, evaluated using different metrics. Standardizing the input variables of the ANN to have a mean of 0 and a standard deviation of 1 is essential. Furthermore, a 5-fold cross-validation was conducted to assess the robustness of the ANN algorithm. The model’s performance, across different configurations of hidden layers and neurons per layer, demonstrates that increasing the number of hidden layers typically enhances accuracy, as illustrated in Table 2. Similarly, increasing the number of neurons in each hidden layer also improves the model’s accuracy. This improvement results from the additional hidden layers or neurons, which increase the connections between neurons, thus optimizing the computation process within the ANN.

The data distribution greatly influences the model’s accuracy, as illustrated in Table 2. Scattered data can reduce the ANN model’s accuracy. To address this challenge, the scattered data were normalized to achieve consistent mean and standard deviation. In addition, the volume of data used in the ANN learning process affects the model’s accuracy. As a result, the XGBoost model was utilized to develop an optimal model for predicting earthquake-induced slope displacements.

The XGBoost algorithm was employed to enhance model accuracy further. A standard XGBoost model with default parameters was used like 100 estimators, γ = 0, λ = 1, and a learning rate (η) of 0.3. The model was evaluated using a repeated 5-fold cross-validation setup, ensuring that data from the same source were not split between training and testing datasets. Cross-validation was conducted using 3-fold cross-validation repeated five times, resulting in a total of 15 folds. The data was normalized using a standard scaler before the process. After each fold, a prediction was generated. To derive a single representative prediction, the predicted values corresponding to each true value were averaged across all folds at the end of the process. All other parameters were set to their default values.

**Fig 5:**
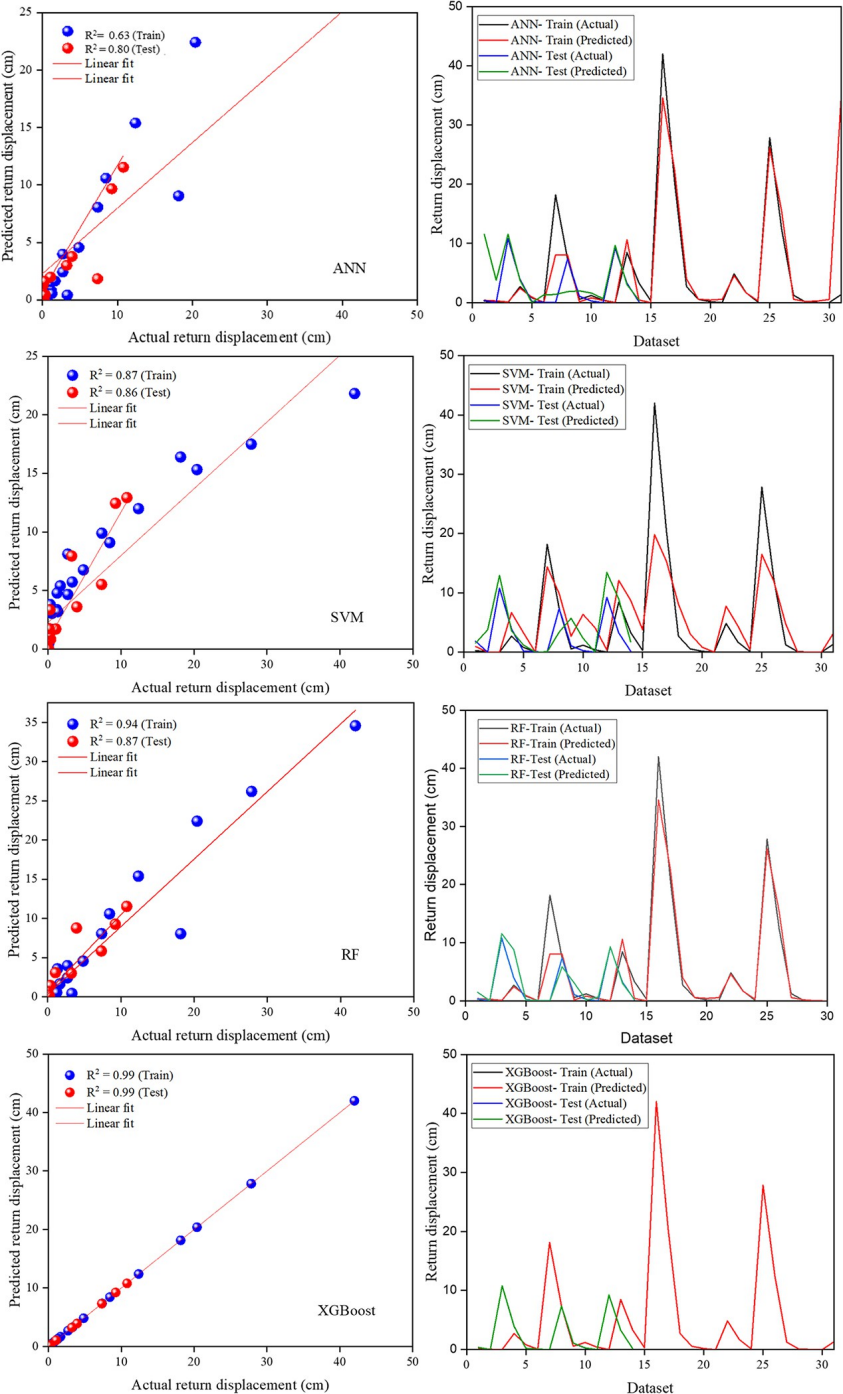
Actual versus predicted return displacement (*u*) by the developed models at the train and test data.

Fig 5 illustrates scatter plots and performance comparisons of actual versus predicted values for displacement (cm) using the ANN, SVM, RF, and XGBoost models on both the train and test data. The R^2^ value for ANN is 0.63 on the train data and 0.80 on the test data. On the other hand, the SVM and RF models achieve an R^2^ values of 0.87 and 0.94 on the train data, and 0.86 and 0.87 on the test data, respectively. Furthermore, the XGBoost model demonstrates an R^2^ value of 0.99 on both the train and test data.

**Fig 6:**
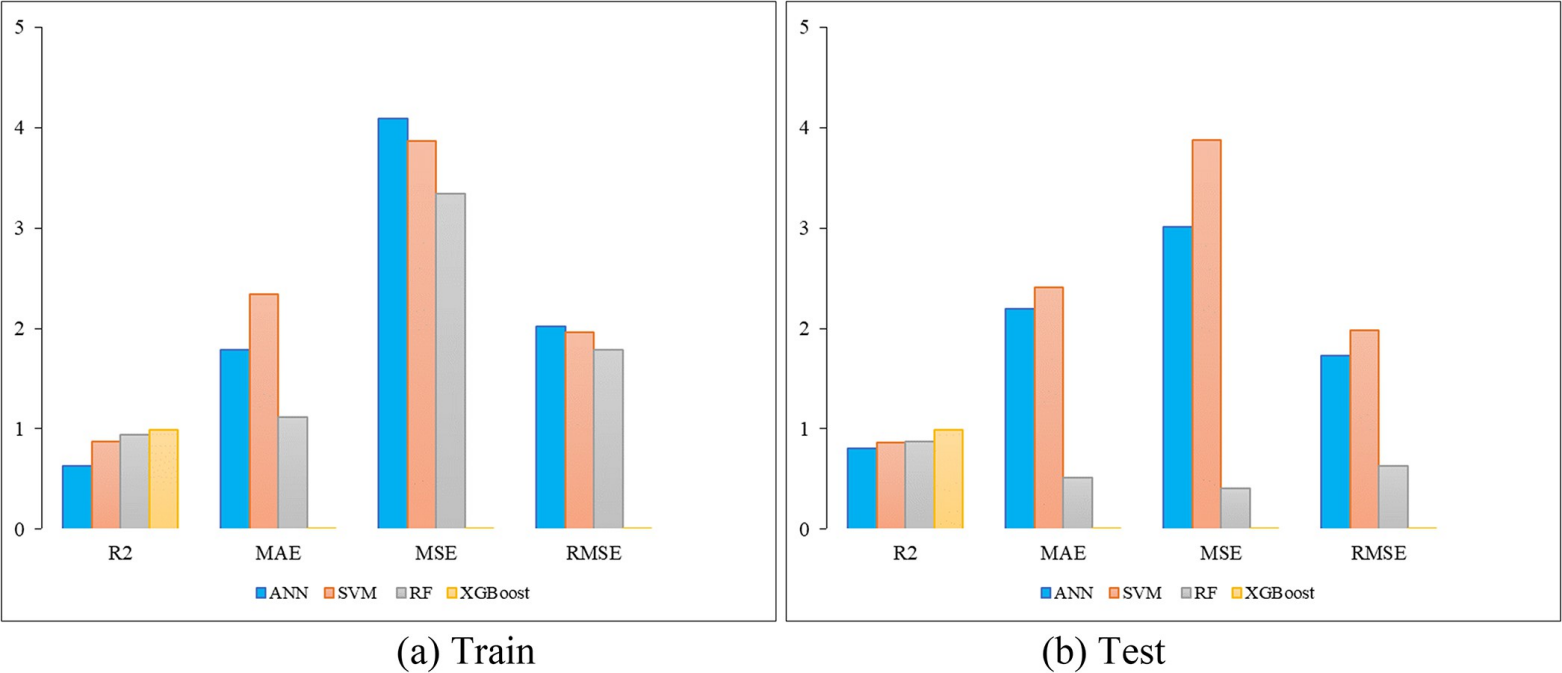
Bar plots of the performance metrics for SVM, ANN, RF, and XGBoost models.

Based on Table 3, the XGBoost model demonstrates the highest scalability and robustness in predicting the return displacement of slopes. Therefore, XGBoost is recommended for predicting the return displacement of slopes induced by earthquakes. Fig 6 depicts the bar plots of the performance metrics for ANN, SVM, RF, and XGBoost models.

**Table 3:** Evaluation criterion performance of developed ANN, SVM, RF, and XGboost models.

Model	Training				Testing			
	R^2^	MAE	MSE	RMSE	R^2^	MAE	MSE	RMSE
ANN	0.63	1.79	4.09	2.02	0.80	2.19	3.01	1.73
SVM	0.87	2.34	3.87	1.96	0.86	2.41	3.88	1.98
RF	0.94	1.12	3.34	1.79	0.87	0.51	0.41	0.63
XGBoost	0.99	0.0017	0.00051	0.00169	0.99	0.00186	0.00068	0.0029

## 5. Sensitivity analysis

**Fig 7:**
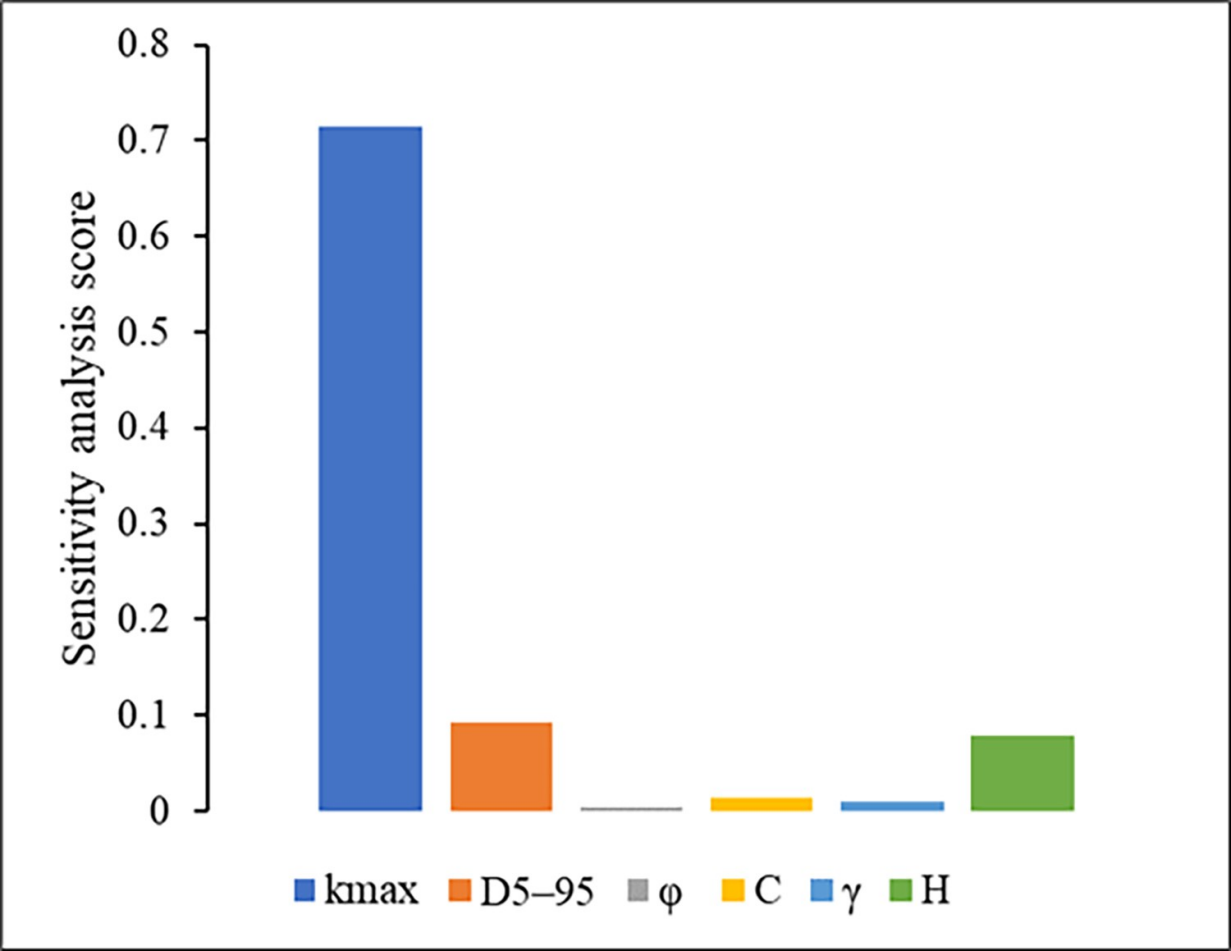
Sensitivity of different attributes in predicting the return displacement.

This study employed ANN, SVM, RF, and XGBoost, to analyze return displacement in earthquake-induced slopes. Among these, XGBoost emerged as the most reliable model. It is important to assess the sensitivity of various parameters for accurate prediction of return displacement in such scenarios. Previous studies have used different methods to evaluate parameter sensitivity [41, 42]. In this context, this study used the XGBoost model to determine the importance of input parameters, as it consistently outperformed ANN, SVM, and RF in predicting earthquake-induced slope displacement. Feature importance techniques assign scores to input parameters based on their effectiveness in predicting the target outcome. The kmax was identified as a critical parameter, with an importance score of 0.714, as illustrated in Fig 7. H also plays a significant role, with an importance score of 0.078. Conversely, input parameters such as C with 0.013, γ with 0.010, D5–95 with 0.093, and φ with 0.0043 were found to be less influential.

## 6. Conclusions

This study developed the ANN, SVM, RF, and XGBoost models to analyze earthquake-induced slope displacement with high accuracy and efficiency. The model’s performance was validated using a dataset from earthquake-induced slope instability analyses and benchmarked against the traditional regression model. Statistical performance indices such as R^2^, MAE, MSE, and RMSE were used to assess predictive accuracy. The ANN model showed an R^2^ value of 0.63 for training and 0.80 for testing, while SVM and RF models obtained 0.87 and 0.94 on training data and 0.86 and 0.87 on testing data, respectively. The XGBoost model outperformed all others model with an R^2^ value of 0.99 on both training and testing data, demonstrating superior robustness in handling complex, nonlinear data.

The XGBoost model provides an effective tool for accurately estimating the tectonic safety of slopes subjected to earthquake-induced displacement. Its ability to serve as a reliable predictive and early warning system offers significant practical applications for slope displacement monitoring. while the model performed well across varying rock conditions, further generalization could be achieved by incorporating more comprehensive slope displacement and geological data. Future work should focus on expanding this model’s application by integrating it into the development of tectonic susceptibility maps.

Although data-driven models typically predict the median displacements of earthquake-induced slopes, XGBoost resilience in managing nonlinear datasets makes it a valuable alternative to deterministic and empirical methods. In the future, the range and number of training samples should be carefully considered to enhance model performance. Advanced machine learning techniques, including hybrid models, could further improve predictive accuracy and offer deeper insights into earthquake-induced slope displacements in future studies.

## Supporting information

S1 File (ZIP)
