# Supplementary figures and images for: Machine learning-based analyzing earthquake-induced slope displacement
Source: PLoS One. 2025 Feb 6;20(2):e0314977. doi: 10.1371/journal.pone.0314977 (PMC11801618; doi:10.1371/journal.pone.0314977)

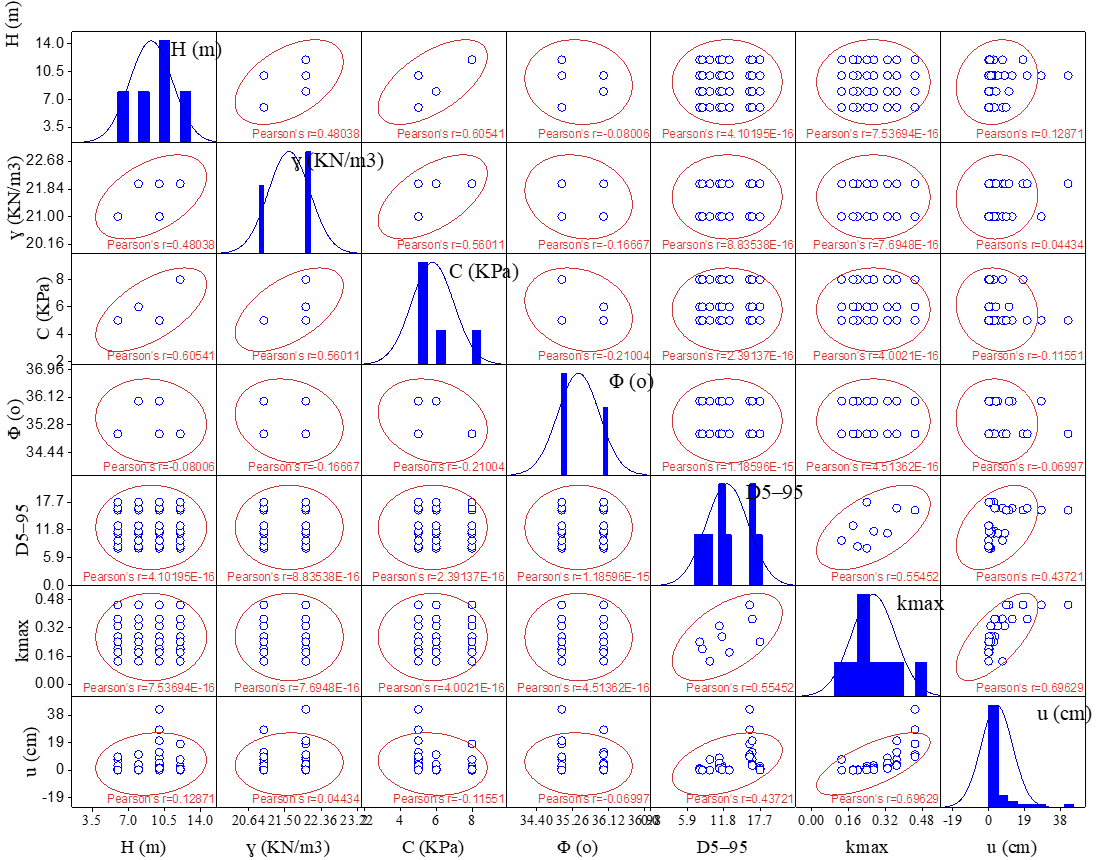

Supplement: S1 File — (ZIP) [file pone.0314977.s001.zip › Supplementary Information/S1-Fig 1.tif]

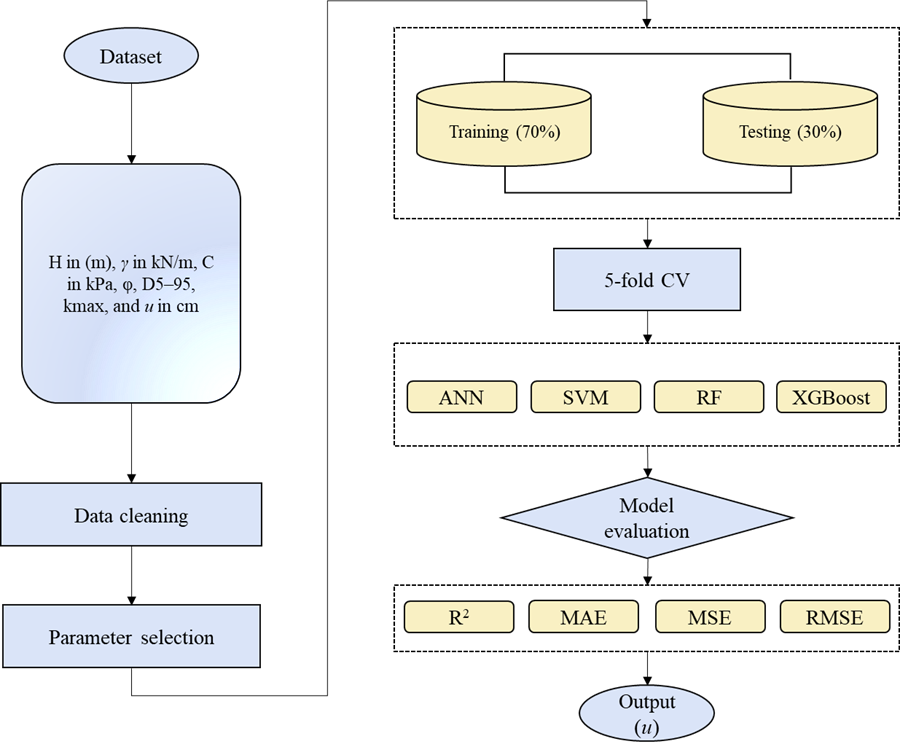

Supplement: S1 File — (ZIP) [file pone.0314977.s001.zip › Supplementary Information/S2-Fig 2.tif]

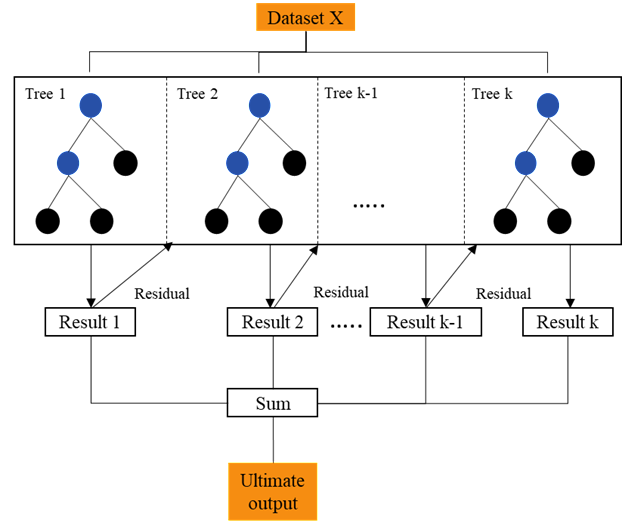

Supplement: S1 File — (ZIP) [file pone.0314977.s001.zip › Supplementary Information/S3-Fig 3.tif]

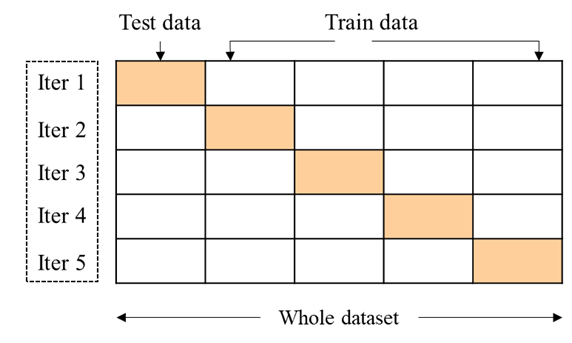

Supplement: S1 File — (ZIP) [file pone.0314977.s001.zip › Supplementary Information/S4-Fig 4.tif]

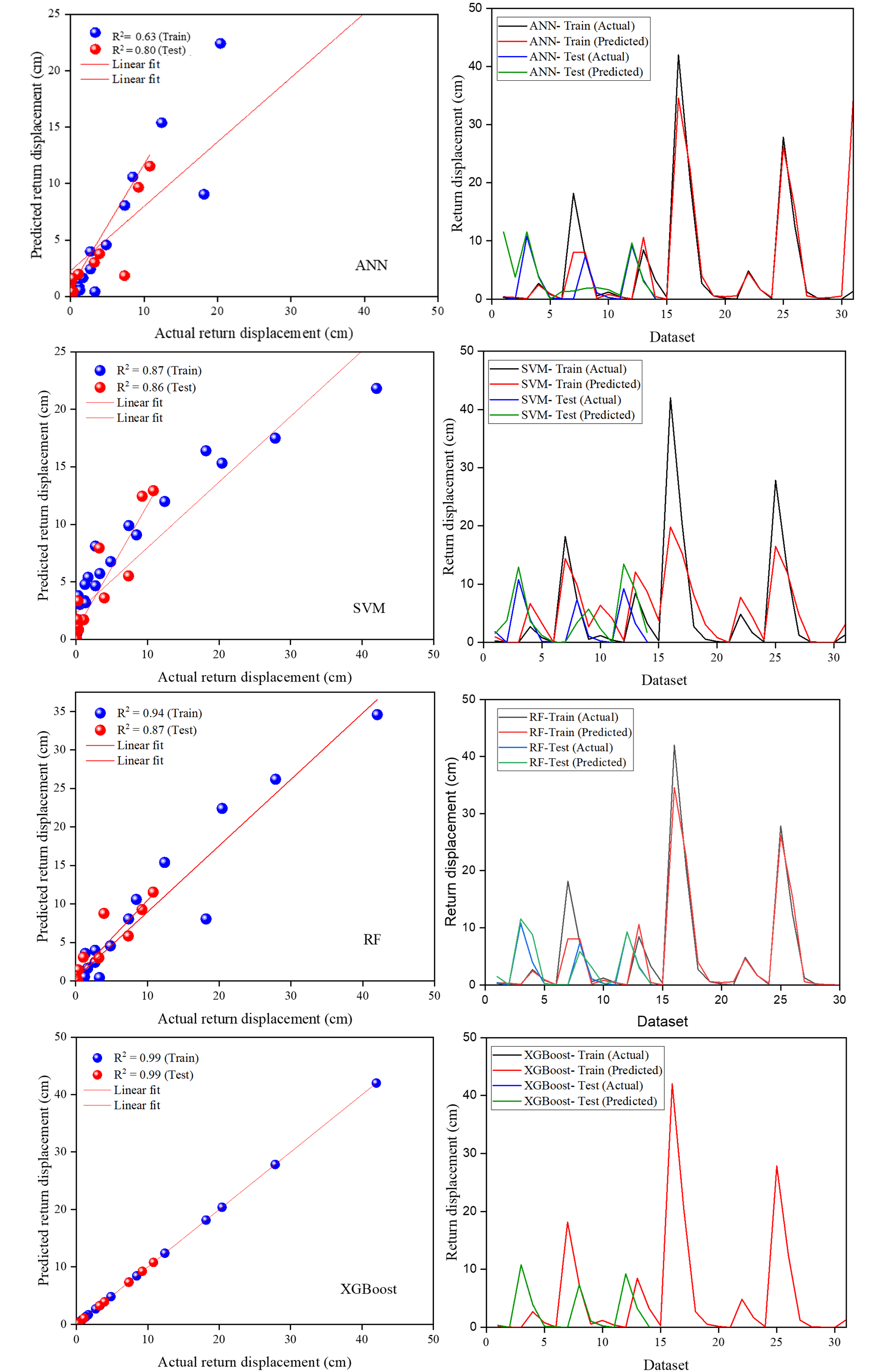

Supplement: S1 File — (ZIP) [file pone.0314977.s001.zip › Supplementary Information/S5-Fig 5.tif]

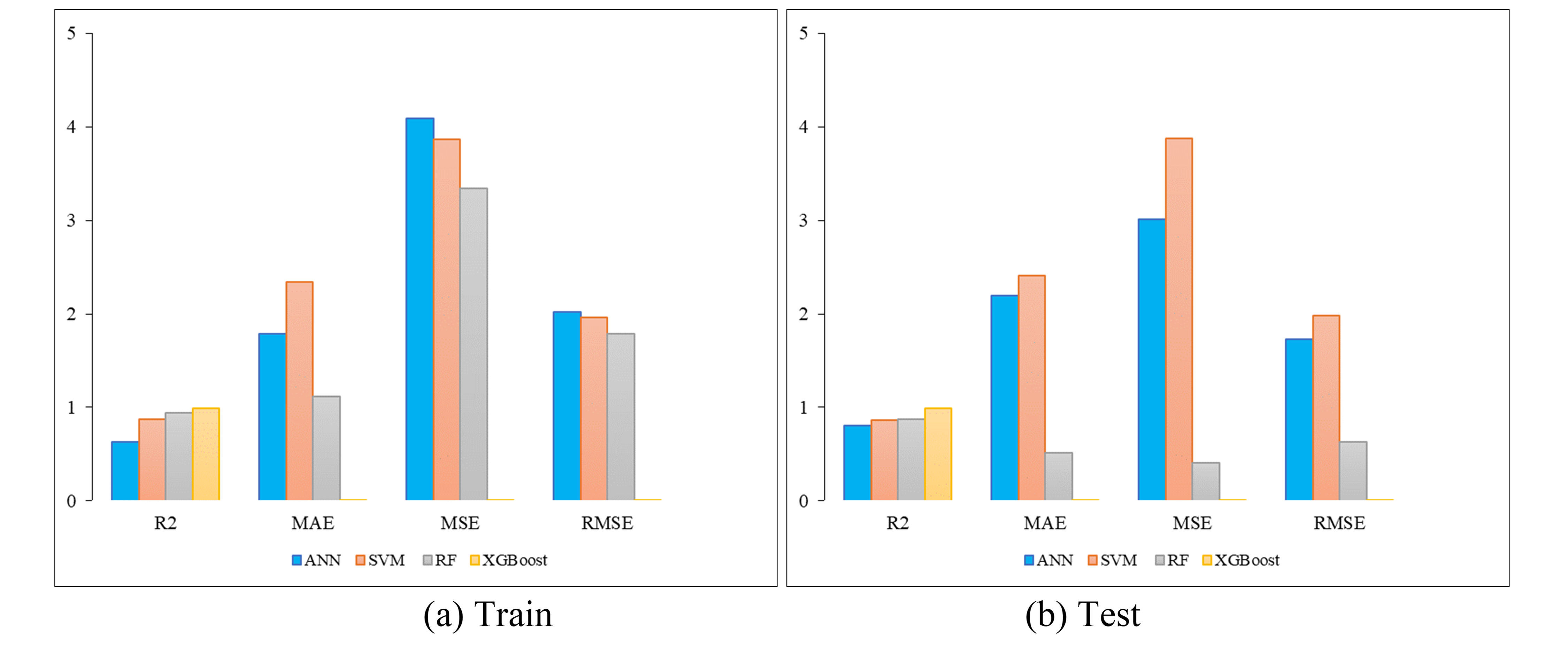

Supplement: S1 File — (ZIP) [file pone.0314977.s001.zip › Supplementary Information/S6-Fig 6.tif]

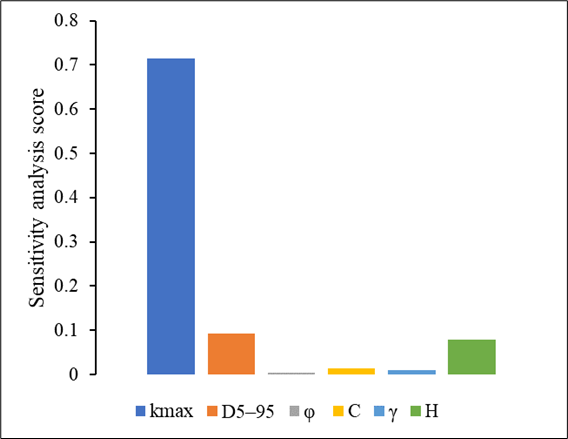

Supplement: S1 File — (ZIP) [file pone.0314977.s001.zip › Supplementary Information/S7-Fig 7.tif]
